# Case of Spontaneous Cure of Spina Bifida, Followed by Hydrocephalus

**Published:** 1884-03

**Authors:** E. Long Fox

**Affiliations:** Consulting Physician to the Bristol Royal Infirmary


### CASE OF SPONTANEOUS CURE OF SPINA BIFIDA, FOLLOWED BY HYDROCEPHALUS.

By E. Long Fox, M.D., F.R.C.P., Consulting Physician to the Bristol Royal Infirmary.

The mother of this patient states that in the middle of the sixth month of pregnancy a furious fowl flew up against the window, at which she was standing, and frightened her so much that she fell to the ground in a sitting posture.

The child was born at full time, and seemed much like other children, except that she suffered from spina bifida, extending from the eighth dorsal to the first lumbar vertebra. There was a slight discharge of clear fluid from the diseased spine. The head was in good proportion to the rest of the body.

After about four months the orifice in the spine became closed over, and no fluid escaped.

When the child was twenty-six months old her state was as follows:—

"The spina bifida, covered in by soft parts, extended from the eighth dorsal to the first lumbar vertebra. From the root of the nose to the occiput the head measured twenty-three inches. From one ear to the other over the vertex twenty.nine inches. The face was small. The eyelids nearly closed. Convergent strabismus. She was unable to raise the head, or to keep it upright when raised by others. She lay always with the head supported. Joints rachitic. No power of standing. No deep reflexes. Involuntary passage of excretions, as in an infant, Mental faculties probably not absent. She showed a distinct liking for music, and seemed to be aware of the presence of her parents. She could move her arms and legs feebly. Sensation to pain good. Swallowed well. Had cut eight teeth. Hair scanty."

In the twenty-ninth month of her age this child died of acute bronchitis. A partial examination was allowed.

"The spinal bones and canal were healthy from above downwards to the eighth dorsal vertebra. Here the spinous processes were absent, and indeed nothing but the bodies of the vertebra remained. The bodies here diverged somewhat to the right. They were rather prominent and formed a lateral bulge. They were very soft and their internal tissue in parts almost grumous. The dura mater spinalis was thickened throughout, and in many places, especially on the anterior surface, was closely adherent to the soft membranes. About the level of the sixth dorsal vertebra the pachymeningitis had caused some constriction of the cord. The soft membranes were somewhat thickened. The cord seemed healthy down to the level of the sixth dorsal vertebra, except that the central canal was large enough to admit an ordinary blowpipe.

"At this spot this central canal was closed. A little below this spot, about the level of the eighth dorsal vertebra, the arrangement of the cord on its posterior aspect showed an almost complete absence of the posterior columns. The central canal was exposed down to the cauda equina, but the constriction at the level of the sixth and seventh dorsal vertebra prevented any communication between this lower portion and the dilated central canal above. The nerves of the cauda equina were matted together in a mass of thickened membranes.

"The medulla oblongata seemed healthy.

"The left hemisphere of the cerebellum was much atrophied. The head measured the same as when seen three months before. The parietal bones were large, but healthy. The frontal sent a long process towards the vertex. The occipital sent a similar process towards the vertex. A very large space at the top of the head was not closed in by bone, but was tented over by a thick fibrous membrane, from which the integument could be easily separated. About the middle of this fibrous tissue, slightly to the right side, the brain and its membrane were adherent to it. The convolutions were flattened, but not otherwise unhealthy. On opening the lateral ventricles nine pints of clear fluid escaped. The third ventricle much enlarged. No granulation of the ependyma of the ventricles. No tubercle. The corpora striata stood out prominently; but all the other basal organs were flattened out beyond recognition."

Very frequently congenital hydrocephalus is associated with congenital hydrorachis. In this case the hydrocephalus was certainly not congenital. From its originating after the cessation of the flow from the bifid spine it was certain during the life of the patient that the fluid came from the central canal. In the face of such a disastrous sequence to nature's efforts for a cure of the congenital malformation, the question of endeavouring artificially to remedy a bifid spine is not free from all anxiety. Such a malformation is not unusually associated with arrest of development in other regions of the nervous centres; and although the enlargement, or rather the insufficient closure of the central canal in such cases is by no means the rule, yet the sequence of events above described is sufficiently common to induce the belief that the co-existence of the two forms of arrested development may materially interfere with surgical success.

